# The pregnancy outcome prediction (POP) study: Investigating the relationship between serial prenatal ultrasonography, biomarkers, placental phenotype and adverse pregnancy outcomes

**DOI:** 10.1016/j.placenta.2016.10.011

**Published:** 2017-11

**Authors:** Francesca Gaccioli, Susanne Lager, Ulla Sovio, D. Stephen Charnock-Jones, Gordon C.S. Smith

**Affiliations:** a Department of Obstetrics and Gynaecology, University of Cambridge, NIHR Cambridge Comprehensive Biomedical Research Centre, Cambridge, UK; b Centre for Trophoblast Research (CTR), Department of Physiology, Development and Neuroscience, University of Cambridge, Cambridge, UK

**Keywords:** Prospective cohort study, Preeclampsia, Fetal growth restriction, Stillbirth, Placental dysfunction, Biomarkers, POP study

## Abstract

Placental dysfunction is implicated in many major complications of pregnancy associated with adverse maternal and infant outcome, such as preeclampsia, fetal growth restriction and stillbirth. Yet, despite years of intensive research, screening for these complications is still largely based upon clinical grounds rather than ultrasonic and/or biochemical assessment of placental function. One of the few widely employed methods for assessment of risk, low first trimester levels of PAPP-A (Pregnancy Associated Plasma Protein A), was identified through secondary analysis of data collected to identify new methods of screening for Down's syndrome rather than as a purposeful search for screening tests for abnormal placentation. Development of improved methods for population screening requires better mechanistic understanding of the pathways leading to placentally-related complications of human pregnancy. This is in addition to a need for identification of biomarkers which reflect the underlying pathology, while predicting associated disease with high sensitivity and specificity. In this paper, we outline some of the challenges and opportunities in this area. Furthermore, we illustrate how some of these can be addressed in research studies using the example of the Pregnancy Outcome Prediction (POP) study, a prospective cohort study conducted in Cambridge, UK.

## 1 Introduction

### 1.1 Clinical context

Pregnancies complicated by preeclampsia (PE) and fetal growth restriction (FGR) are associated with an increased risk of maternal and perinatal morbidity, as well as mortality [1], [2]. FGR is estimated to be the underlying cause in 30–50% cases of stillbirths [3]. Moreover, FGR and PE are defined as placentally-related complications in a large proportion of cases, as they are often characterized by defective placental development (e.g. inadequate re-modelling of maternal spiral arteries and altered uteroplacental blood perfusion) [4]. Therefore placental dysfunction is one of the underlying factors which contribute to PE and FGR, and consequently stillbirth. This offers a great opportunity to identify women at risk using biomarkers reflecting defective placentation.

**Fig. 1:**
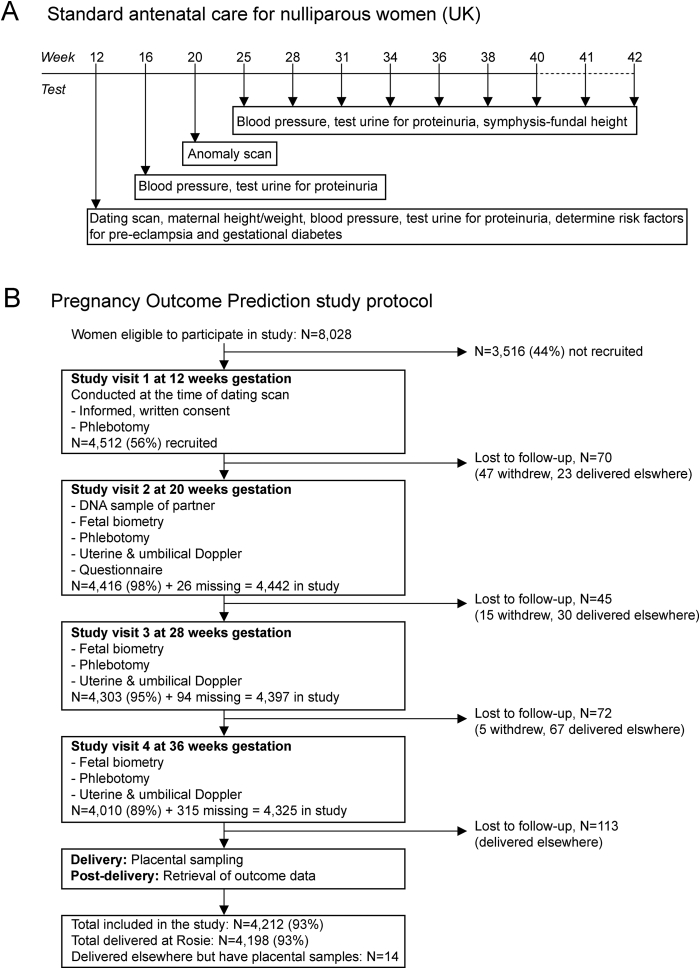
A) Standard antenatal care for nulliparous women in the UK, including 10 routine midwife visits and additional visits for women delivering after 40wkGA. B) Flow chart of the POP study.

Currently, antenatal care of first pregnancies in UK involves 10 routine midwife visits (Fig. 1A). Such high intensity reflects a poor discrimination of risk: most severe adverse outcomes in first pregnancies occur to women who are deemed to be “low risk” at booking. Moreover, the primary screening method for women is for the midwife to measure symphyseal-fundal height with a tape measure, check blood pressure and test urine for proteinuria. The only widely used biomarker for placentally-related complications (low first trimester PAPP-A, see below) was the result of secondary analysis of Down's syndrome screening studies. An alternative to the current approach is personalised antenatal care, where the pattern and frequency of visits is proportionate to the given woman's risk of key complications, as estimated by the patient's clinical records, medical imaging and/or biomarkers. This could avoid unnecessary intervention in healthy women and facilitate the targeting of resources in a more cost-effective and clinically effective manner for high risk women. We are attempting to address this goal by conducting a prospective cohort study of unselected nulliparous women, focusing on serial ultrasonography measurements and collection of biological samples. In this article we outline rationale and conduct of the study, while addressing some general points for researchers considering similar studies.

### 1.2 Clinical screening using placental assessment

Fetal growth is evaluated using ultrasonographic fetal biometry and calculation of estimated fetal weight (EFW). Placental function is assessed using Doppler flow velocimetry of the maternal uterine and fetal umbilical arteries. Flow resistance of the uterine artery decreases during the first half of pregnancy due to invasion of the maternal vessels by the trophoblast cells. Persistence of high resistance patterns of flow in both the uterine and umbilical artery in mid-gestation and in the third trimester of pregnancy has been associated with altered placental function and structure as well as increased risk of obstetric complications [5], [6], [7], [8]. Currently, these methods are applied selectively to women with pre-existing risk factors or acquired complications of pregnancy. However, the sensitivity of selective use of ultrasonography for detecting small babies is poor, in the region of 20–25% [9], [10]. The obvious alternative, universal ultrasonography, is not recommended: systematic reviews have failed to show an improvement in clinical outcome with universal ultrasonography in late pregnancy [11]. However, the apparent absence of benefit may be due methodological weaknesses [12], such as study heterogeneity, failure to design an effective interventional strategy after the identification of the screen-positive cases, and lack of statistical power [13]. Although there is no currently strong case for population-based screening using universal ultrasound, this has been identified as a high priority for further research [14].

Currently in the UK, clinical guidelines recommend increased surveillance of women having low first trimester levels of the placentally-derived protein PAPP-A [15], [16]. However, this association was found following secondary analyses of Down's syndrome screening studies, which demonstrated that low maternal levels of PAPP-A in the first trimester were associated with PE, FGR and stillbirth risk [17], [18]. More recently other biochemical biomarkers related to angiogenesis (i.e., Placental Growth Factor (PlGF), soluble fms-like tyrosine kinase 1 (sFlt-1), soluble Endoglin (sEng)) have been proposed as potential screening tests for PE and FGR [19], [20], [21], [22]. Although potentially promising, their clinical usefulness has been shown to be modest in several studies [19], [23], [24]. Their diagnostic effectiveness as a screening test remains to be properly assessed in low risk populations, close to term (when an effective intervention is available, i.e. delivery of the baby), in association with different phenotypes of the disease (especially in pregnancies with small babies), and in combination with other biomarkers.

## 2 Designing a study for identification of novel screening appraoches

### 2.1 Overview, aims and design of POP study

The Pregnancy Outcome Prediction (POP) study is a prospective cohort study of nulliparous women attending the Rosie Hospital (Cambridge, UK) for their dating ultrasound scan [25]. The study included 4512 women with a viable singleton pregnancy; study participants provided written informed consent and were recruited between January 2008 and July 2012 (Fig. 1B) [10]. The rationale behind recruiting only nulliparous women is that they have a higher absolute risk of developing pregnancy complications compared to multiparous women [26], [27]. Furthermore, they lack previous pregnancy outcome information which is an important risk indicator for subsequent pregnancies [28], [29].

The POP study aimed both at evaluating performance of known biomarkers and serial ultrasonography in assessing maternal and fetal well-being, as well as identifying novel biomarkers. The study is sufficiently large to be powered for relatively uncommon adverse pregnancy outcomes (Table 1) [25]. Women attended four study visits scheduled every 8 weeks, starting with the first trimester. Participants had blood taken during the dating/recruitment visit (at approximately 12 weeks gestation (wkGA)), as well as at three subsequent visits (at ∼20wkGA, ∼28wkGA and ∼36wkGA). On each of these three visits research ultrasound scans were also performed to assess fetal biometry, amniotic fluid index, uterine and umbilical uterine artery Doppler flow velocimetry and placental maturity. Serial measurements and sampling offer the chance to monitor the parameters of interest throughout gestation, and changes across pregnancy have been suggested to have more predictive power than single snapshots in time [30], [31]. The final evaluation at 36wkGA was intended as a means to screen for risk of complications at term, when effective intervention to prevent placentally-related complications of pregnancy is available (i.e., early delivery). The timing of this visit was informed by previous analyses demonstrating that tests of placental dysfunction (i.e., biochemical and ultrasonic screening) have a better predictive value when performed close to disease onset [32]. Patients and clinicians were blinded to these ultrasonography results, unless major congenital abnormalities, placenta praevia, severe oligohydramnios or breech presentation (36wkGA only) were detected. At the 20wkGA visit, a questionnaire was completed to retrieve the patient's demographic data and medical history. During the same visit a DNA sample of the partner and measurements of the partner's height/weight were collected. After delivery, biopsies of the placenta, placental membranes, umbilical cord, and cord blood were collected (see below).

**Table 1:** Numbers and rates of adverse outcomes of women participating in the POP study.

Outcome	N = 4212
GDM	192 (4.6)
Missing	6 (0.1)
**Pre-eclampsia/hypertension**	
De novo PE, not severe	53 (1.3)
De novo PE, severe	93 (2.2)
Superimposed PE, not severe	85 (2.0)
Superimposed PE, severe	42 (1.0)
GH, not severe	62 (1.5)
GH, severe	23 (0.6)
Essential HT	99 (2.4)
Missing	5 (0.1)
**sPTD**	113 (2.7)
Missing	41 (1.0)
**Induction of labour**	1355 (32)
**Mode of delivery**	
Spontaneous vaginal	2051 (49)
Assisted vaginal	992 (24)
Intrapartum caesarean section	726 (17)
Pre-labour caesarean section	424 (10)
Missing	19 (0.5)
**Outcome of birth**	
Livebirth	4161 (99)
Miscarriage (pre-viable <24wks)	13 (0.3)
Termination of pregnancy <24wks	17 (0.4)
Termination of pregnancy ≥24wks	1 (0.0)
Stillbirth	11 (0.3)
Neonatal death	6 (0.1)
Missing	3 (0.1)
**Any neonatal morbidity**	292 (6.9)
Metabolic acidosis	45 (1.1)
5-min Apgar <7	42 (1.0)
Neonatal unit admission	240 (5.7)
Severe adverse perinatal outcome	39 (0.9)

Abbreviations: GDM = gestational diabetes mellitus, PE = preeclampsia, GH = gestational hypertension, essential HT = essential hypertension, sPTD = spontaneous preterm delivery (23-36 completed weeks, <23 weeks included in the missing category). Data were obtained from examination of the clinical case records and by linkage to the hospital’s electronic databases and are expressed as n (%). Any neonatal morbidity was defined as one or more of the following: delivery with metabolic acidosis (cord blood pH <7.1 and base deficit >10mmol/L), a 5-minute Apgar score <7, or admission to neonatal unit at term (within 48 hours from birth for at least 48 hours). Severe adverse perinatal outcome was defined as one or more of the following: stillbirth (not due to congenital anomaly), neonatal death at term (not due to congenital anomaly), hypoxic ischaemic encephalopathy at term, use of inotropes at term, mechanical ventilation at term, severe metabolic acidosis at term (defined as pH <7.0 and base deficit >12 mmol/L). For characteristics that have no “missing” category, data were 100% complete.

A further point of note is that the population in the POP study is low risk and homogeneous, from a demographic and socio-economic perspective (Table 2). The low risk nature of the cohort is an important study strength from the perspective of both evaluating screening tests and identifying novel mechanisms/biomarkers of placental dysfunction. It is well-recognized that studies can overestimate the positive predictive value of a screening test by extrapolating results of selected and high risk cohorts to the general population (called the “spectrum effect”) [33]. Moreover, when studying mechanisms it would clearly be problematic if cases were largely derived from a population of different ethnicity and socio-economic status than controls. This is a potential hazard as many placentally-related complications of pregnancy are more common in minority and socio-economically deprived communities.

**Table 2:** **Characteristics of the POP study cohort**. Hospital record data comparing women recruited to the POP study (Cambridge, UK) with eligible women who were not recruited.

Characteristic	Recruited N = 4265	Not recruited N = 2909	P Value
**Age, years**			
<20	115 (2.7)	166 (5.7)	<0.0001^a^
20–24.9	527 (12)	550 (19)	
25–29.9	1210 (28)	876 (30)	
30–34.9	1637 (38)	901 (31)	
35–39.9	668 (16)	353 (12)	
≥40	108 (2.5)	63 (2.2)	
**BMI, kg/m**^2^			
<25	2339 (55)	1634 (56)	0.10
25–29.9	1009 (24)	629 (22)	
30–34.9	330 (7.7)	198 (6.8)	
35–39.9	106 (2.5)	86 (3.0)	
≥40	60 (1.4)	33 (1.1)	
Missing	421 (9.9)	329 (11)	
**White ethnicity**	3914 (92)	2490 (86)	<0.0001
Missing	62 (1.5)	46 (1.6)	
**Smoker at booking**	347 (8.1)	350 (12)	<0.0001
Missing	188 (4.4)	141 (4.9)	
≥1 previous miscarriage (1st trimester)	540 (13)	297 (10)	0.003
≥1 previous miscarriage (2nd trimester)	108 (2.5)	57 (2.0)	0.15
**Mode of delivery**			
Vaginal	2094 (49)	1537 (53)	0.0002
Assisted vaginal	1006 (24)	702 (24)	
Caesarean section	1165 (27)	670 (23)	
**Gestational age, weeks**			
Preterm: <24	22 (0.5)	18 (0.6)	0.29^a^
Preterm: 24-<33	43 (1.0)	32 (1.1)	
Preterm: 33-<37	156 (3.7)	119 (4.1)	
Term: ≥37	4012 (94)	2727 (94)	
Missing	32 (0.8)	13 (0.4)	
**Birth weight centile**			
SGA (<10th)	390 (9.1)	298 (10)	0.13
Severe SGA (<3rd)	102 (2.4)	94 (3.2)	0.03
Missing	55 (1.3)	33 (1.1)	
**Transfer to neonatal unit**	216 (5.1)	176 (6.1)	0.07
Missing	34 (0.8)	18 (0.6)	
**Outcome of birth**			
Livebirth	4231 (99)	2889 (99)	0.75
Miscarriage	5 (0.1)	3 (0.1)	
Termination of pregnancy	18 (0.4)	12 (0.4)	
Stillbirth	11 (0.3)	4 (0.1)	
Missing	0 (0)	1 (0.03)	

Abbreviations: BMI = body mass index, SGA = small for gestational age. Data are expressed as median (inter-quartile range) or n (%) as appropriate. *P*-values are for difference between groups calculated using the two-sample Wilcoxon rank-sum (Mann-Whitney) test for continuous variables and the Pearson Chi-square test for binary and categorical variables.

a Score test for trend of odds is reported for categorical ordered variables if the trend is approximately linear. For fields where there is no category labelled “missing”, data were 100% complete. Maternal age is defined as age at delivery. Missing category is not included in statistical tests. The hospital's delivery database (PROTOS) was used to compare basic characteristics of women recruited to the study and eligible women who were not recruited. Recruited N = 4512 minus 247 who delivered elsewhere and had no PROTOS record = 4265. Not recruited N = 3516 eligible minus 607 without PROTOS record = 2909. Modified from Sovio et al. (2015) [10].

### 2.2 Other prospective cohort studies: NuMoMs2b and SCOPE

Other prospective cohort studies recruiting nulliparous women with singleton pregnancies have been conducted, such as the *Nulliparous Pregnancy Outcome study*: *Monitoring Mother-to-be* (NuMoMs2b) and the *Screening for Pregnancy Endpoints* (SCOPE) [34], [35]. Approximately 10,000 women were recruited in the NuMoMs2b study in multiple centres across the United States. The SCOPE study was a multicentre cohort study including about 5500 women in Australia, Ireland, New Zealand, and the United Kingdom. As in the POP study, women enrolled in the SCOPE and NuMoMs2b studies were recruited in early pregnancy, at ∼12-16wkGA. Although the cohort populations are similar in the three studies, there are some important differences. For instance, in the SCOPE and NuMoMs2b studies the last study visits were conducted at ∼24wkGA and ∼28wkGA, respectively, therefore excluding a collection point close to term which may be important for predicting adverse pregnancy outcome [32]. Additionally, placental samples were collected from a small percentage of the participants in the NuMoMs2b study and the SCOPE study did not include routine placenta collection. Finally, all three studies performed research ultrasound examinations. However, the results of these scans were reported in both SCOPE and NuMoMs2b, whereas in POPs the results were blinded in >94% of cases. One of the criteria for a Level 1 study of diagnostic effectiveness is blinding results from the screening test being evaluated, in this case serial universal ultrasonography [10].

### 2.3 Multiple methodological approaches around a single study

Since the POP study is a large prospective study, it offers the possibility of analysing parameters and values measured at a specific gestational, but also changes over time. This has been suggested to be useful in the search of biomarkers for pregnancy complications [30], [31]. Moreover, multiple approaches are available for sample and data analysis, which can be carried out on the whole study cohort or subsets of participants. Alternative methodological approaches can be used, such as case-cohort study and nested, matched case-control study.

#### 2.3.1 Prospective cohort study

When using this type of analysis, data available from all the study participants (such as ultrasound scan results and maternal demographic information) are employed to calculate incidence rates, relative risks, and confidence intervals [36]. This approach has been used to compare the detection rates of small for gestational age (SGA) infants using selective and universal ultrasound [10]. Here, blinding of the research ultrasonography was crucial and this is, to our knowledge, the only large scale Level 1 study of the diagnostic effectiveness of universal ultrasound as a screening test for SGA. Several other papers on results from the POP study have been published using this methodology (details below).

#### 2.3.2 Case-cohort study

A case-cohort methodological approach utilises all cases (of differing types; i.e., PE, gestational diabetes, FGR) within the cohort and a randomly selected sub-cohort as control group [37]. Thus for all differing adverse outcomes there is one common group of controls. Such an approach may be advantageous if expensive or labour intensive assays are being performed, since it reduces the number of samples processed while still achieving a similar precision compared to using measurements from the whole cohort. Similar to whole cohort analysis, calculation of relative risks is possible using a case-cohort methodological approach. This design is currently being used in the POP study to investigate metabolomic markers of a range of pregnancy outcomes.

#### 2.3.3 Nested matched case-control study

With a nested case-control methodological approach, identified cases within a larger cohort are selected and matched one-to-one with healthy controls [36]. Cases are paired with controls based upon key characteristics (e.g., maternal body mass index (BMI), gestational age (GA), and fetal sex) and statistical comparisons are based on paired methods (e.g., paired *t*-test for continuous variables and the McNemar test for dichotomous variables). Therefore, potential confounders are adjusted through matching. The nested case-control approach is particularly useful for molecular studies where methodologies are very expensive and/or labour intensive, as well as when adjustments using multivariate statistical models are not applicable. In the POP study there are currently several projects with this design, based on Next Generation Sequencing (NGS) and histological examinations of placental specimens (discussed below).

#### 2.3.4 Synergies between study designs

A key element of the POP study is that data used for one element of the analysis can strengthen other aspects of the analysis. For example, the results of uterine and umbilical Doppler flow velocimetry were employed in the analysis of universal ultrasound as a screening test for SGA and FGR. The same data then allows SGA infants to be phenotyped in greater detail. We can show that better phenotyping of SGA cases results in a much larger number of positive results in RNA-Seq analysis (see below). We thereby conclude that heterogeneity within groups with apparently similar outcomes can mask differences between cases and controls.

## 3 Current findings

Several projects planned with samples from the POP study are currently ongoing: the analysis of clinical records and outcome data parallels and complements the wet lab work.

### 3.1 Ultrasonographic fetal biometry

Historically, fetal biometry measurements had a particular focus upon the identification of SGA babies [38]. As described above, clinically indicated ultrasonography in the third trimester has a low sensitivity (20–25%) for detecting SGA babies [9], [10]. We demonstrated that screening of nulliparous, low risk women with universal third trimester fetal biometry triples the detection of SGA infants [10]. Moreover, analysis of the abdominal circumference (AC) growth velocity was the most effective method for discriminating between healthy SGA infants and those with FGR.

Other recent analyses of the POP study cohort have yielded insights from serial assessment of fetal biometry. We have reported that reduced fetal growth velocity of the femur between 20 and 28wkGA was associated with a ∼2.5 fold increased risk of spontaneous preterm birth [39]. We have also reported associations with excessive growth. We found that maternal diagnosis of gestational diabetes mellitus (GDM) at or after 28wkGA was preceded by excessive AC growth between 20 and 28wkGA; GDM effects on fetal growth were additive with the effects of maternal obesity [40]. Given that biochemical testing for GDM typically takes place at around 28wkGA, data indicate that earlier screening and intervention may result in better control of excessive fetal growth.

### 3.2 Automated immunoassay analysis of maternal serum samples

**Fig. 2:**
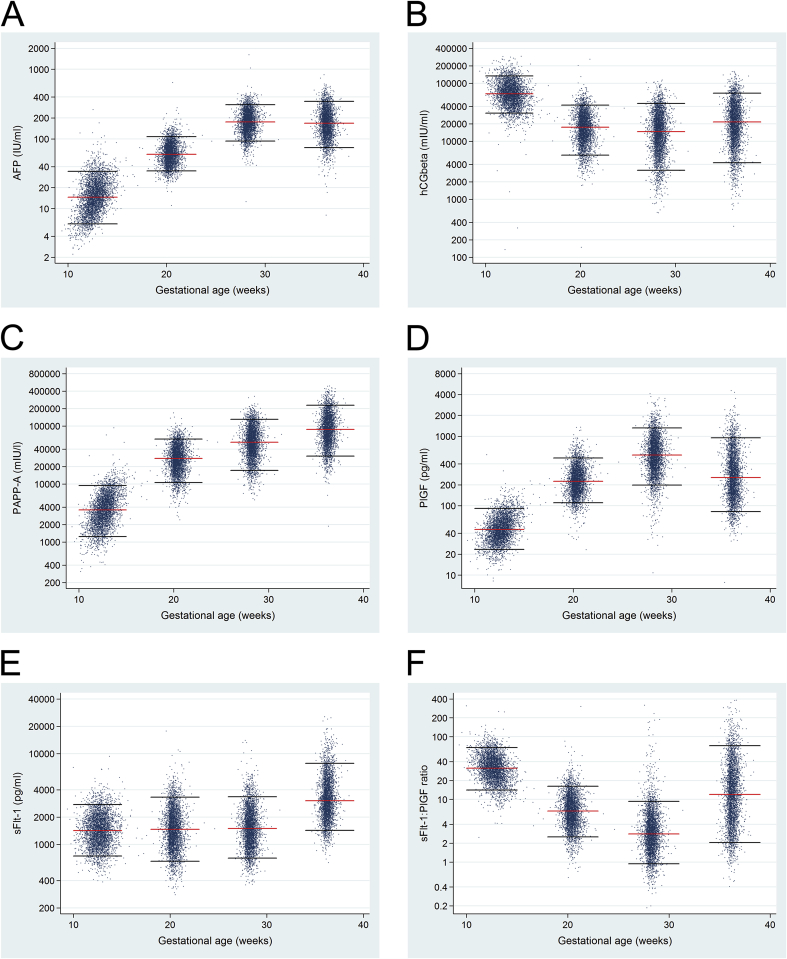
Biomarkers measured in maternal serum samples from women completing the study (n = 4212). A) AFP, B) hCG, C) PAPP-A, D) PlGF, E) sFlt-1, and F) the ratio of sFlt-1/PlGF were measured in serum samples collected at ∼12wkGA (n = 4078), ∼20wkGA (n = 4021), ∼28wkGA (n = 3994), and ∼36wkGA (n = 3780). Samples were analysed using clinical grade Roche Elecsys immunoassays on an e411 platform (Roche). The red lines indicate the 50th percentile values and the black lines represent the 5th and 95th percentile values at 12, 20, 28 and 36 completed weeks of GA ± 2 weeks difference.

Although maternal blood is an easily accessible and attractive resource of potential biomarkers for pregnancy complications, the factors studied so far revealed low predictive power for PE and FGR. The maternal serum samples collected in the POP study at ∼12wkGA, ∼20wkGA, ∼28wkGA and ∼36wkGA have been analysed to determine whether combination of universal ultrasound and previously described placental biomarkers may improve identification of pregnancies at increased risk of adverse outcome. A total of 15,874 serum samples from the 4212 women who completed the study have been analysed using clinical grade Roche Elecsys immunoassays on an electro-chemiluminescence platform (cobas e411 analyzer, Roche Diagnostics, Mannheim, Germany). We analysed levels of proteins where there is a clinical grade assay available and where previous studies have shown associations with placental function: alpha fetoprotein (AFP), chorionic gonadotropin (hCG), PAPP-A, PlGF, and sFlt-1. All the samples have been processed and the analysis in relation to the pregnancy outcomes is currently ongoing. Interestingly, a plot of the raw data demonstrates that the 5 proteins show different patterns of change in relation to gestational age, suggesting that each reports different aspects of placental function (Fig. 2).

### 3.3 Placental morphometry and histology

**Fig. 3:**
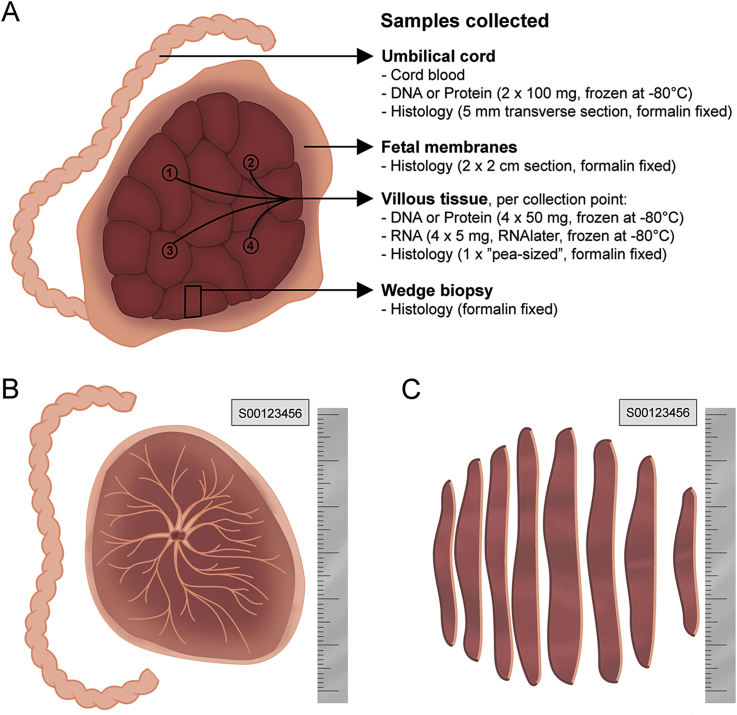
Overview of placental sampling. A) Tissue biopsies were collected from umbilical cord, fetal membranes, and villous tissue. For the villous tissue, four collection points were selected and the samples from each collection point stored separately. Samples for DNA or Protein were snap frozen in liquid nitrogen and then stored at −80 °C. Biopsies for RNA extraction were soaked in RNAlater overnight (4 °C) and then frozen in liquid nitrogen and stored at −80 °C. All samples for histology were fixed in formalin for 24 h (4 °C) before being embedded in paraffin wax. A full thickness wedge biopsy was collected from the edge of the placenta. If the placenta was processed more than 30 min after delivery, RNA and cord blood samples were not collected. B) After processing the tissue biopsies, the fetal membranes and the umbilical cord were trimmed off and photographs of the placenta were taken, both of the maternal side (basal plate) and fetal side (chorionic plate). C) Lastly, each placenta was cut along its longest diameter into 1 cm thick sections with the fetal side facing upward. The sections were tipped, exposing the cut surface (fetal side to the right and maternal side to the left) and a photograph was taken. In each photograph a ruler and a label with a unique sample ID number were included.

A series of photographs were taken of maternal and fetal sides of the whole placenta, as well as 1 cm thick strips (Fig. 3) [https://www.obgyn.cam.ac.uk/research/pops-2/]. Using these images, the relation between prenatal ultrasonic measurements and postnatal placental morphometry has been investigated [6]. We demonstrated that reduced placental surface area is associated with high resistance uterine artery Doppler at 20wkGA, while lower placental weight is associated with high resistance umbilical Doppler at 36wkGA. Furthermore, reduced placental surface area and placental weight are associated with reduced fetal growth velocity.

Several ongoing projects are based upon histopathological analysis of placental biopsies. For example, we are currently studying matched case-control pairs, focusing on both PE and FGR. 3420 biopsies from 855 placentas have been fixed and cut for microscopy (Fig. 3). Each biopsy is analysed using nine stains (hematoxylin and eosin (H&E), 5-hydroxymethylcytosine, CD14, CD3, CD31, CD4, CD79α, CD8, and neutrophil elastase), for a total of 30,780 biopsies stained using the fully automated platform BOND-III (Leica Biosystems). High-resolution images are generated by an Aperio AT2 scanner (Leica Biosystems). These are analysed quantitatively and blind to clinical outcome using a Visiopharm computer assisted stereology toolbox and image analysis. H&E sections are also being reported by two perinatal pathologists who are blind to outcome.

### 3.4 Next Generation Sequencing (NGS)

NGS is one of the major methods of analysis employed in studying placental biopsies, with a focus both on the transcriptome and the epigenome (small RNAs, DNA methylation and histone modifications). These analyses have the following aims: (i) understanding the mechanisms leading to placentally-related complications, (ii) studying the association between pregnancy complications and the presence of infectious agents in the placenta, and (iii) identifying of novel biomarkers that can be evaluated in the matched blood samples. This broad approach has previously yielded novel markers. Using older expression array-based technology, it was discovered that excess placental production of sFlt1 contributes to increased maternal circulating sFlt-1 in pregnancies complicated by PE [41]. Circulating sFlt-1 measurements, combined with PlGF levels, have been shown to be useful in the assessment of PE. High sFlt-1 concentrations and increased sFlt-1:PlGF ratio have been measured in women who later developed PE five weeks before the clinical onset of the disease [42], [43]. The same authors reported that an high sFlt-1:PlGF ratio is highly predictive of PE when tested in combination with soluble endoglin levels. Moreover, sFlt-1:PlGF ratio has now been shown to be clinically useful in ruling out preeclampsia within one week after the test when the condition is suspected [44].

In the POP study, a total of 320 placental samples (from 55 matched severe SGA [<5th percentile]/appropriate for gestational age (AGA) pairs, and 105 matched PE/control pairs) have been studied using RNA-Seq analysis of both long RNAs (i.e., totRNA-seq identifying mRNAs and lncRNAs) and small RNAs (i.e., smallRNA-seq identifying miRNAs and piRNAs). This work has generated large volumes of data. For example, in the projects based on totRNA-Seq, we have an average of 102 million reads (125 base pair long, single end) per sample, resulting in a total of ∼33 billion reads. The differentially expressed RNAs or the corresponding coded proteins, in case of altered mRNAs, will be later studied in the maternal circulation. In these NGS-based projects, information derived from the clinical records and outcome data collected in the POP study were used not only to identify and characterise disease subtypes, but also to carefully match cases and controls (e.g., according to GA, maternal BMI and age, smoking, fetal sex, labour, and mode of delivery). The nested case-control design has a higher statistical power than unmatched designs and it accounts for confounding through matching, as described above. Interestingly, the initial analysis of the results from the SGA/AGA pairs revealed that the accurate definition of clinical subgroups of SGA (i.e., small healthy babies or small babies with poor AC growth velocity, SGA pregnancies with high resistance umbilical Doppler, high resistance uterine Doppler, low maternal PAPP-A, or maternal hypertension) led to the identification of a higher number of differentially expressed RNAs compared to an analysis including all the SGA samples. This finding illustrates the importance of deep phenotyping of cases when the outcome can be the end point of multiple different pathological processes.

## 4 Conclusions

**Fig. 4:**
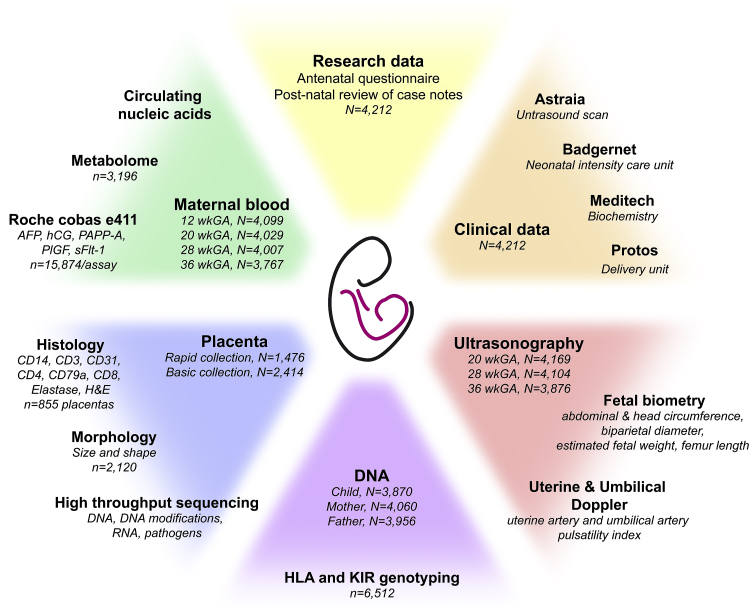
The POP study at a glance. Each coloured area includes clinical data/measurements or samples collected during the study and their corresponding results/projects. Patients' clinical data are managed using electronic databases of ultrasonography (Astraia, München, Germany), neonatal intensive care (Badgernet, Clevermed Ltd, Edinburgh, UK), biochemical tests (Meditech, Westwood MA, USA) and delivery (Protos, iSoft, Banbury, UK). Approximately 230,000 blood and tissue samples are stored in six −80 °C freezers (with empty backup freezer, backup power supply, telephone alert system and automatic triggering of liquid CO2 to maintain the temperature at −65 °C in the event of failure). All samples are labelled with identifiers and unique 2D barcodes and tracked using Pro-curo software (Pro-curo Software Ltd, Horsham, UK). N indicates the number of patients from whom the samples/records were obtained; n indicates the number of samples analysed. Abbreviations: HLA = human leukocyte antigen, KIR = Killer-cell immunoglobulin-like receptor.

Careful design of a large prospective study involves accurate collection and storage of clinical information, outcome data and biological samples (Fig. 4). Clinical information helps defining the biological samples and, likewise, the results obtained from the biological samples contribute to accurate phenotyping of the patient and the pregnancy. Moreover, such a design allows the identification of clinically useful biomarker candidates. These considerations need to be taken into account when designing a study ultimately aiming at improving management of patients and pregnancy outcomes.

## Conflict of interest statement

G.C.S. receives/has received research support from GE (supply of two diagnostic ultrasound systems used in the current study). Other commercial interests for G.C.S. are as follows: support from Roche (supply of equipment and reagents for biomarker studies, ∼£600,000 in value) and from GlaxoSmithKline (GSK) (∼£200,000) for a project to study effects of retosiban in human myometrium, payment to attend advisory boards by GSK and Roche, and payment for consultant work for GSK. G.C.S. is named inventor in a patent submitted by GSK (U.K.) for novel application of an existing GSK compound for the prevention of preterm birth (PCT/EP2014/062602). No other potential conflicts of interest relevant to this article were reported.
